# Embedded Palladium Activation as a Facile Method for TiO_2_-Nanotube Nanoparticle Decoration: Cu_2_O-Induced Visible-Light Photoactivity

**DOI:** 10.1002/open.201200041

**Published:** 2013-01-10

**Authors:** Anca Mazare, Ning Liu, Kiyoung Lee, Manuela S Killian, Patrik Schmuki

**Affiliations:** [a] Department of Materials Science and Engineering, WW4-LKO, University of Erlangen-NurembergMartensstrasse 7, 91058 Erlangen (Germany) E-mail: schmuki@ww.uni-erlangen.de

**Keywords:** annealing, copper deposition, palladium activation, TiO_2_ nanotubes, water splitting

In the present work, we introduce a facile and reliable approach to decorate TiO_2_ nanotubes homogenously with well-separated copper oxide nanoparticles. For this, we use a palladium-containing titanium alloy (commercial Ti 0.2 atom % Pd) and grow anodic, self-organized oxide nanotubes. Palladium is embedded in the tube wall during growth and acts as activator for a subsequent electroless copper-nanoparticle deposition at distinct sites within the nanotubes. Using an appropriate heat treatment, the copper nanoclusters can be converted to Cu_2_O or CuO. Such decorated nanotubes show a significant enhancement of the water-splitting performance under artificial solar light irradiation.

Over the past decade, TiO_2_ nanotube layers formed by a self-organizing anodization process have attracted tremendous interest in science and technology,^1^ as their geometry and properties have in the past demonstrated and continue to promise better performance in applications such as photocatalysis, solar cells, or biomedical coatings.^2^–^8^ One of the highly active research fields is their application for photocatalytic reactions including the decomposition of numerous pollutants (e.g., CO_2_),^4^–^6^ and the direct or photoelectrochemical splitting of water into H_2_ and O_2_.^9^–^12^ For water-splitting applications, TiO_2_ is generally considered only to be efficient under photoelectrochemical conditions (applied bias) and, due to a band gap of 3.0–3.2 eV, under UV illumination. Efforts to overcome these drawbacks are mainly based on band-gap engineering of TiO_2_, or coupling TiO_2_ with a p-type narrow-gap semiconductor with suitable band edge positions. In this context, a main candidate is Cu_2_O, as it is potentially able to form a visible light-active p–n junction. Therefore, considerable efforts are undertaken trying to optimally decorate TiO_2_ and TiO_2_ nanotubes with Cu_2_O particles or clusters,^13^–^16^ with the target to obtain a homogeneous distribution of Cu_2_O particles over the TiO_2_ nanotubes in order to reach an efficient performance. When trying to achieve a homogeneous deposition with conventional electrodeposition processes, a frequent difficulty is that particle deposition takes place on the top^14^, ^16^ of the TiO_2_ nanotubes rather than uniformly within the nanotube structure.

In the present work, we report a novel approach to uniformly decorate TiO_2_ nanotube walls with copper oxide nanoparticles. The key is to grow oxide nanotubes from a palladium-containing titanium alloy (in our case, commercial Ti0.2Pd). This alloy was selected, as palladium is well-known for its activation of electroless deposition reactions, for example, of copper or nickel.^17^, ^18^ After anodic tube formation from the alloy, palladium is embedded in the oxide of the nanotube wall and can be exploited as activation site for selective and defined electroless copper deposition. The copper particles can then be thermally converted to Cu_2_O or CuO, and the resulting structures show significantly enhanced activity for visible-light water-splitting reactions.

Figure 1 shows the morphologies of the nanotube (NT) layers grown anodically on a plain titanium metal sheet (Ti-NT, see Figure 1 A) and on the TiPd alloy (TiPd-NT, see Figure 1 B). In both cases, the oxide nanotube layers were grown in the same glycerol/water electrolyte, and in both cases identical tube morphology with typical tube lengths of approximately 850 nm and tube diameters of about 80 nm is obtained. These layers were then annealed and immersed in a copper plating solution; Figures 1 C, E and 1 D, F show scanning electron microscope (SEM) images of the tube layers on titanium and TiPd, respectively, after the deposition process. Clearly, in the case of the palladium-containing alloy, copper deposits can be formed regularly over and within the tube layers (Figure 1 D), whereas for the reference TiO_2_ nanotubes obtained on titanium no trace of copper deposits could be observed (Figure 1 C).

**Figure 1:**
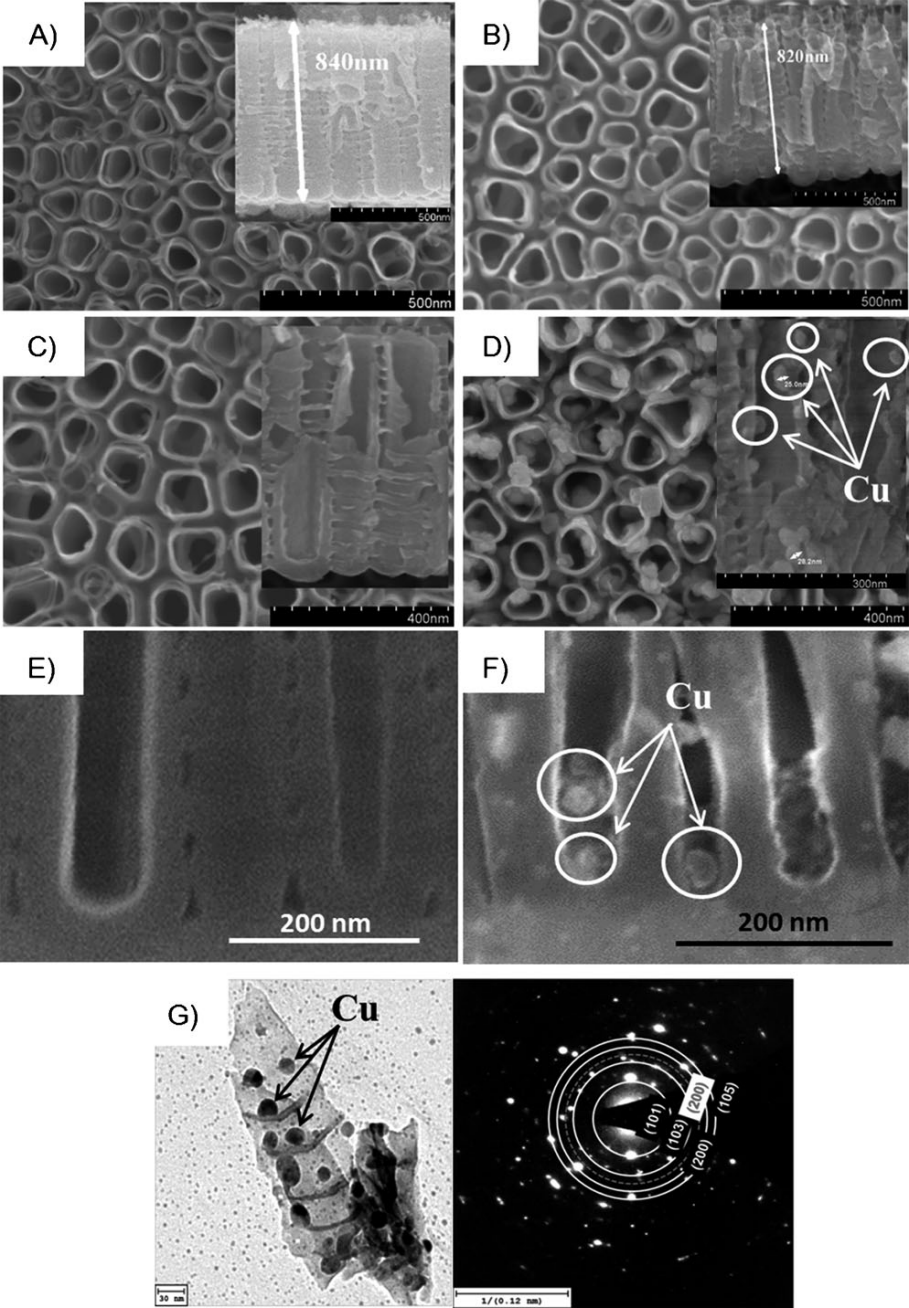
SEM top-view and cross-sectional-view (inset) images. A) TiO_2_ nanotubes on Ti (Ti-NT); B) TiO_2_ nanotubes on (TiPd-NT); C, E) Cu deposition on Ar-annealed TiO_2_ nanotubes on Ti (Ti-NT/Cu); D, F) Cu deposition on Ar-annealed TiO_2_ nanotubes on TiPd (TiPd-NT/Cu) [E and F are ion-milled cross-sections]; G) TEM image of TiPd-NT/Cu, thermally treated at 140 °C, with the TEM-SAD patterns.

The transmission electron microscope (TEM) image in Figure 1 G for the tubes in Figure 1 D shows that copper was deposited in the tubes, and that after annealing Cu_2_O particles had a size of approximately 25 nm. After annealing the copper-decorated tubes in air at 140 °C, TEM selected area diffraction (TEM-SAD) patterns (Figure 1 G) are in line with the formation of Cu_2_O. Experiments with or without annealing treatments and with different copper deposition conditions show that for non-annealed TiPd samples, copper deposition occurs in a much less defined manner, and the timing of the electroless process needs to be optimized to avoid an overgrowth of the copper deposit. However, it should be noted that the argon-annealing step was extremely crucial for obtaining a good distribution of copper deposits on and into the tubes, thus being very important for a reproducible and reliable electroless deposition.

From the X-ray diffraction (XRD) patterns in Figure 2, one can deduce that for conditions as in Figure 1 D, metallic copper is deposited. For reference, XRD measurements were also carried out on Ti-NT (Figure 2 A) and TiPd-NT (Figure 2 B) after argon annealing. From the corresponding patterns, it is obvious that after the argon annealing, for both substrates, TiO_2_ is present in the anatase form. XRD patterns for copper-deposited samples after thermal oxidation at 140 or 300 °C in air show complete conversion of the copper to copper oxide. The XRD data in Figure 2 D indicate that the treatment at 140 °C leads to conversion to Cu_2_O (main peak at 36.3°), while 300 °C annealing leads to conversion to CuO (main peak at 35.5°, see Figure 2 E). These findings are in line with X-ray photoelectron spectroscopy (XPS) data that show the presence of CuO at 300 °C (peak at 933.7 eV, see Figure 2 F), while the peak at 932.5 eV corresponding to Cu_2_O or copper has entirely vanished (separation of Cu_2_O from Cu is not conclusively possible in XPS).

**Figure 2:**
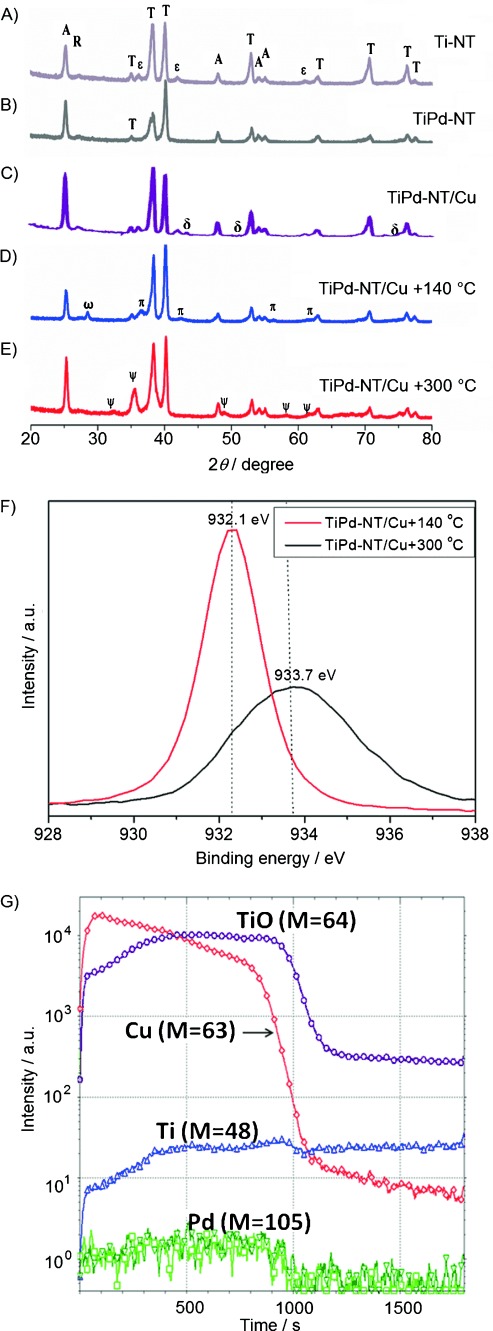
XRD patterns for the annealed TiO_2_ nanotube layers. A) Ti-NT; B) TiPd-NT; C) TiPd-NT/Cu; D) TiPd-NT/Cu, thermally treated at 140 °C; E) TiPd-NT/Cu, thermally treated at 300 °C; T=titanium, A=anatase, R=rutile, ε=TiO, δ=Cu, π=Cu_2_O, ψ=CuO, ω=copper sulfide (contamination). F) XPS spectrum of the sample with Cu deposition on Ar-annealed TiO_2_ nanotubes, thermally treated at 140 and 300 °C; G) TOF-SIMS analysis of TiPd-NT/Cu, thermally treated at 140 °C.

To evaluate the distribution of copper species over the nanotube length, we acquired a time-of-flight secondary ion mass spectrometry (TOF-SIMS) sputter depth profile (Figure 2 G). It is apparent that copper and palladium can be detected uniformly distributed over the entire tube layer thickness (please note that due to matrix effects, the sensitivity to an element changes from oxide to substrate).

To evaluate the activity of the different structures in solar light water-splitting devices, we performed photocurrent measurements under AM 1.5 (air mass coefficient; 100 mW cm^−2^) conditions. The photocurrent response shown in Figure 3 clearly demonstrates that both CuO and Cu_2_O lead to a significantly enhanced positive photoresponse compared with the plain TiO_2_ nanotubes. Cu_2_O leads to a significantly higher photocurrent than CuO.

**Figure 3:**
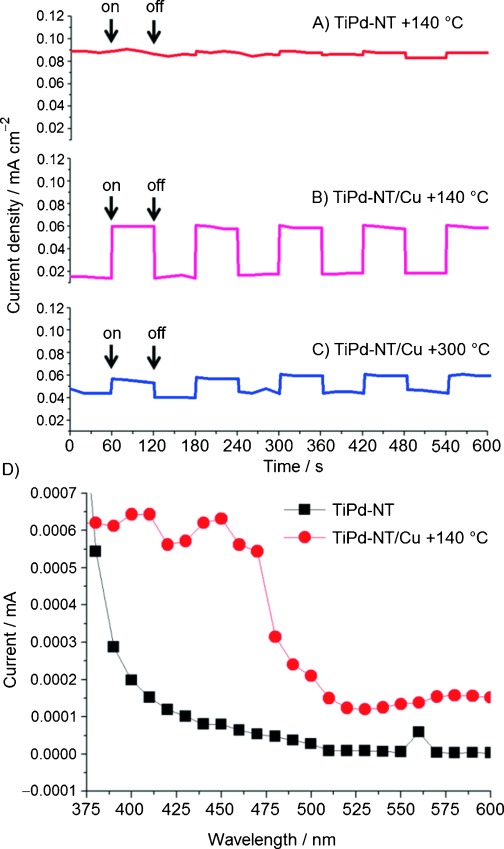
A–C) Photocurrent transients obtained by using an electrometer and a two-electrode configuration in 1 m Na_2_SO_4_ (buffered at pH 4.9), under simulated AM 1.5 illumination provided. D) Photocurrent spectra recorded at +100 mV vs. Ag/AgCl in 1 m Na_2_SO_4_ (pH 4.9).

To clearly illustrate the photoactivity in the visible range, we measured photocurrent spectra in the same solution as used for the AM 1.5 measurements (Figure 3 D). The data are in line with reports on Cu_2_O electrodes (e.g., P. E. de Jongh, D. Vanmaekelbergh, J. J. Kelly^19^).

Overall, the present work demonstrates a novel, direct and efficient way to decorate TiO_2_ nanotubes successfully with copper, Cu_2_O and CuO particles. The work shows that anodic oxidation of TiPd alloy can lead to active palladium species embedded in the TiO_2_ nanotube walls. This allows triggering specific initiation sites for electroless copper deposition along the tube walls. This sensitization approach may not be limited to TiO_2_/Pd and copper deposition but may find much wider applications (e.g., Pd sensitization for Ni,^20^ Cu,^21^ Ag^22^). In the present case, we show that tubes functionalized with Cu_2_O clearly lead to enhanced photoactivity, thus providing potential for application in direct photolytic water splitting.

It may be considered that Cu_2_O in the course of use is oxidized to CuO,^23^ which would reduce its efficiency as a material establishing a p–n junction. Nevertheless, the fact that 25 nm Cu_2_O or CuO nanoparticles with a band gap energetically negative to TiO_2_^24^ establish a band offset in either case can be beneficial to charge carrier separation.

## Experimental Section

Well-ordered TiO_2_ nanotubes were obtained from a TiPd alloy with a Pd content of 0.2 atom % and a sample thickness of 1 mm, by electrochemical anodization in glycerol/H_2_O (60:40, *v*/*v*) with 0.5 wt % NH_4_F at a potential of 20 V for 2 h. The setup and other parameters used for self-organized tube formation are the same as previously described for pure Ti.^25^, ^26^

The resulting oxide nanotubes were annealed in a furnace in argon at 450 °C for 1 h. The deposition bath for electroless Cu deposition consisted of CuSO_4_⋅5 H_2_O (0.7 g), KNaC_4_H_4_O_6_⋅4 H_2_O (2.5 g) and HCOH (2.5 mL) for a total of 100 mL solution. The solution was adjusted to pH 12.5 with NaOH. Cu deposition was carried out by immersing the nanotube layers in the plating solution for 90 s at RT. The samples were then thermally treated in a JetFirst 100 rapid thermal annealer (Jipelec, Montpellier, France) at different temperatures (140 and 300 °C) in air with heating and cooling rates of 30 °C min^−1^ to transform the metallic Cu particles to oxide. The nature of the thermal treatment after Cu deposition is very important due to the fact that thermally treating Cu either leads to Cu_2_O and/or CuO.^27^–^29^

For morphological characterization of the TiO_2_ nanotubes, a S4800 field emission scanning electron microscope (FE-SEM; Hitachi) was used. X-ray diffraction (XRD) patterns were collected using an X′pert PMD diffractometer (Philips) with a X′celerator detector (Panalytical, Almelo, The Netherlands), using graphite-monochromatized CuKα radiation (*λ*=1.54056 Å). Further morphological and structural characterization was carried out with a CM 30 TEM/STEM (Philips). The chemical nature of Cu was obtained from X-ray photoelectron spectroscopy (XPS) spectra using a PHI 5600 XPS surface analysis system (Physical Electronics, Chanhassan, MN, USA). In order to investigate the depth distribution of Cu and Pd, time-of-flight secondary ion mass spectrometry (TOF-SIMS) negative depth profiles were recorded on an Ion-TOF SIMS V instrument (ION-TOF, Münster, Germany) in dual beam mode. A pulsed 25 keV Bi^+^ liquid-metal ion beam bunched down to <0.8 ns was used for spectra generation and a 2 keV Cs^+^ ion beam for sputter removal of the samples. Signals were identified according to their isotopic pattern as well as exact mass, calibrated to CH_2_, C_2_, CN and CNO signals, and Poisson correction was employed.

Open-circuit photocurrent measurements were carried out using an 616 Digital Electrometer (Keithley, Cleveland, OH, USA) and a two-electrode configuration^30^ (Pt as cathode) under simulated AM 1.5 (100 mW cm^−2^) illumination provided by a solar simulator (300 W Xe with optical filter; Solarlight, Glenside, PA, USA) in 1 m Na_2_SO_4_ (buffered at pH 4.9 with potassium hydrogen phthalate and NaOH). Photocurrent transients under applied bias were recorded at +100 mV vs. Ag/AgCl in the same buffered 1 m Na_2_SO_4_ solution, using a three-electrode setup.
